# Regressive Saccades During Reading: Effects of Target Distance and the Role of Individual Differences in Spatial Memory and Linguistic Skill

**DOI:** 10.3390/jemr19040082

**Published:** 2026-08-03

**Authors:** Anne Friede, Christian Vorstius, Albrecht Inhoff, Ralph Radach

**Affiliations:** 1 Division of General and Biological Psychology, University of Wuppertal; Max-Horkheimer-Str. 20, 42119 Wuppertal, Germanyradach@uni-wuppertal.de (R.R.); 2 Psychology Department, Binghamton University, Binghamton, NY 13902-6000, USA

**Keywords:** spatial memory, re-reading, regressive saccades, regression strategies

## Abstract

The current study examined the control of long-range regressive eye movements during sentence reading. Skilled readers were asked to read single-line sentences for comprehension. As a secondary task, they identified a probe word presented to the right of each sentence, and then went back to check the corresponding target word for spelling errors that had been added after reading. The regression target was located either close to or far from the probe. Consistent with prior work, initial regressions were larger for far than for near targets, and the finding of far targets required more eye movements and more search time. We replicated previous descriptions of visuomotor regression strategies but also discovered a novel strategy in which readers frequently send their initial regressions to the center of the line, followed by subsequent saccades. Apparently, this happened primarily when spatial knowledge on saccade target location was lacking. Spatial memory and reading skill made distinct contributions to regression targeting. Memory skills strongly determined primary regressions, especially to near targets, whereas reading speed influenced the time needed to attain targets with subsequent saccades.

## 1. Introduction

Reading is a complex skill that involves the coordination of linguistic information extraction with visual processing and oculomotor control (see [1] for review). As the constituents of text are spatially ordered, eye movements must follow this order for the acquisition of new linguistic information. Not all eye movements (saccades) progress with word order, however, and a considerable proportion, approximately 15–25%, are directed at prior words in the text (e.g., [2]). These backward-directed saccades (regressions) range considerably in size, and large regressions can extend across several prior words or lines in the text. There is converging evidence that medium- or long-range regressions are primarily executed when readers encounter lexical and syntactic processing difficulties, and that readers regress to prior text to reprocess it (e.g., [2,3,4,5,6,7]).

How do readers move the eyes to a previously read word when it is relatively far from the current viewing location, and thus outside the range of effective vision? Two accounts have been offered. One, the spatial coding hypothesis, maintains that readers endow the represented linguistic form of an identified word with a spatial tag, and then use this tag, possibly with reference to a visible landmark (such as page margins) to direct long-range regressions to the regression target [8,9,10]. The other, the verbal reconstruction hypothesis, maintains that accessible linguistic representations are used to reconstruct the location of prior words in the text [11,12].

According to the spatial coding hypothesis, readers should be able to direct regressions with a relatively high degree of accuracy at a retrieved page and line location. Testing of this prediction requires control over the execution of regressions and the location of regression targets. In their pioneering work, Kennedy and Murray [9] achieved this control by presenting a probe word after the reading of single-line sentences that either was part of the sentence or was a close synonym. Participants had to decide (yes/no), whether the probe had occurred in the sentence. With this approach, the regression target in a sentence was often reached with a single highly accurate regression (single shot). This occurred, irrespective of whether the word was near the end of the sentence, and therefore close to the probe word, or near the beginning of the sentence, and therefore relatively far from the probe. Occasional corrective saccades that followed some regressions were rather small (averaging 2.7-character spaces), indicating that readers could reach regression targets with high spatial precision, irrespective of target distance. According to the spatial coding hypothesis, this occurred because word identification had included a spatial tagging of words, and these spatial tags could be used later to guide the eyes to corresponding—previously identified—linguistic content (see also [8] for a discussion).

Additional support for the spatial coding hypothesis was obtained in experiments that manipulated the apparent spatial location of the regression target [8]. To generate the illusion of a spatial change, single-line sentences were shown inside rectangular frames. In two shift conditions, the frames were moved to the left or right by two-character positions when the eyes moved from the sentence to the probe word, creating an apparent shift in the sentence toward or away from the probe word location, although the location of the sentence remained unchanged. In spite of the frame shift, the size of the regression to the target was not changed, presumably because the spatial index for the target was established when the word was identified and thus prior to the frame shift. Inhoff, Weger & Radach [13] used a more direct test of spatial coding by shifting a sentence prior to the execution of a short-distance regression, again with the result that this manipulation did not affect the amplitude of regression saccades.

In these experiments, data selection of regressions was often confined to a narrow band of landing positions near the target, and this could have exaggerated the accuracy of regressions. In more recent work without such selection constraints, regressions were still spatially selective, being larger when directed to far than to near targets, but the regression error was relatively large, up to 17-character spaces, and readers typically programmed multiple corrective regressions [12]. That is, an initial saccade moved the eyes typically toward but not onto the target, and the initial regression was followed by a subsequent search for the target. Similarly, Mitchell et al. ([14], Experiment 2), who examined regressions in response to syntactic garden pathing, showed that regressions were rarely directed at the area that caused the misanalysis of the sentence, and that readers typically used two to three consecutive saccades to reach it.

When effects of spatial distance were pitted against linguistic distance (the number of words in-between a denoted regression target and the location from which the regression was launched), type of distances influenced the size of regressions from the source location toward the regression target [12]. This implied that regressions were controlled by more than one type of knowledge, and Weger and Inhoff proposed a hybrid model in which initial regressions were guided by spatial knowledge while subsequent regressions were guided by verbal/linguistic knowledge.

Using a similar approach, Guérard et al. [15,16] showed, however, that an articulatory suppression task (articulating the letters A, B, C, D repeatedly with a duration of 500 ms per letter) diminished the accuracy of initial regressions, although the effect was confined to relatively specific conditions. In Guérard et al. [16], where participants read two-line sentences with and without articulatory suppression, suppression increased the initial regression error and the number of corrective saccades for regressions initiated from the second line to the first line, but not the targeting of regressions from the end of the first line to the beginning of the first line. These results can be taken to suggest that, under some conditions, the spatial coding hypothesis may need to be put on its head: regressions may sometimes be guided primarily by non-spatial representations.

The programming of regressions is likely to vary from reader to reader, and these individual differences can be exploited to understand the types of information that control the targeting of regressions. Hyönä et al. [17] clustered competent readers into four groups with distinct eye movement characteristics. Only one group relied on the frequent use of regressions during the reading of a short story segment. This group appeared to write the best story summaries and had the highest working memory span.

Inhoff et al. [18] found that the ability to reprocess sentences was strongly associated with successful comprehension of text. They grouped oculomotor measures into two types and reprocessing (consisting of regressions and re-reading time) was directly linked to accuracy of text reading. Moreover, a cluster analysis assigned slow readers into two groups. Only the ones who used regressions achieved good reading comprehension despite their slow reading speed, emphasizing the importance of regressions for successful reading.

Taken together, prior work has suggested that readers represent the spatial location of words together with extracted linguistic information. Spatial indexes of words are then used to guide regressions towards corresponding linguistic content. In addition, linguistic knowledge can also serve to guide regressions to a prior location in the text. While regressive saccades can be large, even crossing several lines, the vast majority of regressions extends across no more than one or two words, and these regressions are directed with a relatively high degree of accuracy at the center of targeted words [19]. Linguistic knowledge could therefore be used primarily when the finding of the regression target is difficult, as may occur when it is far from the launch point of a regression segment [11], when a sentence is unparseable [14], or when readers have to perform a potentially demanding secondary task [16].

The present work consists of two parts, one involved the reading of one-line sentences followed by a regression back to a specific target word and the second consisting of the assessment of readers’ spatial and dynamic working memory capacity and of their general text comprehension skill. With respect to the first part of the work, the primary goal was to provide the first comprehensive quantitative analysis of visuomotor strategies used to move back to previous locations during reading.

To this end, we started from Murray and Kennedy’s [10] procedure that required participants to read one-line sentences followed by a probe word to the right of each sentence. Participants were asked to make a yes/no decision about whether the probe had been part of the sentence. This task often led to correct responses without the need to execute a regressive saccade. We therefore added a meaningful secondary task, asking readers to check target words (indicated by the probe at the end of the sentence) for spelling errors, which were present in 50 percent of all cases. Importantly, the spelling errors were inserted after the sentence had been read for the first time by crossing an invisibly boundary at the sentence end. To examine the role of saccade target distance, target words were located either relatively near a current fixation, or somewhat further away. In both cases, the linguistic embedding of target words was carefully controlled (see methods).

In line with a large body of literature on goal-directed saccades, and, more specifically, the literature on regression during reading [5], we expected that regressions to near targets would be more successful in terms of initial accuracy, number of saccades and time needed to attain the target. We also expected to replicate the findings by Kennedy et al. [8] and Inhoff et al. [13] who used saccade contingent shift manipulations, further supporting the view that the information used for the programming of regression saccades primarily stems from spatial memory rather than the visual processing of presaccadic configurations. With respect to specific visuomotor strategies, we hypothesized to find substantial proportions of very accurate regressions as well as sequences of backtracking saccades, corresponding to the regression patterns observed in the work of Kennedy and colleagues. In addition, we suspected that we might see cases of participants moving to the beginning of sentences and then executing forward saccades, as described in Frazier and Rayner’s [7] classic work on regression strategies after the reading of garden path sentences. Our idea was to quantify the frequency with which theses different visuomotor strategies were used, primarily to evaluate how often traces in spatial memory are reliable enough to support accurate long-range regressions.

As stated above, the second goal of our work was the assessment of individual differences in spatial memory capacity and reading skill in order to examine the utilization of spatial and linguistic knowledge for the control of regression strategies. To this end, we used pre-existing individual differences in readers’ linguistic and spatial abilities to determine whether these abilities influenced regression guidance (see also [11]). Our approach distinguished two types of spatial skills. One using representations for fixed (static) spatial locations, and another using representations for continuously changing spatial locations (dynamic). The use of landmarks to code spatial word locations, as assumed by the spatial coding hypothesis, would rely on the use of static spatial knowledge, as the position of a target vis-à-vis a landmark will remain fixed throughout sentence and passage reading. However, the eyes keep moving away from an identified word, and the position of the word relative to this current fixation location changes continuously. The distinction between spatial memory types is also consistent with the view that spatial working memory encompasses functionally distinct spatial abilities [20]. We intended to determine which aspect of spatial ability, static or dynamic spatial memory, is more relevant for patterns of regression during reading.

In addition to spatial abilities, readers’ text comprehension skill was also assessed. We expected that the role of these language skills would be noticeable, but relatively modest in comparison to spatial abilities, in line with results by Weger and Inhoff [12]. These authors looked at the accuracy of regressions within two-line passages and found that, when pitted against each other, actual spatial distance was dominant over linguistic distance (in terms of number of words to the target).

According to Weger and Inhoff’s hybrid model, spatial memory acts to guide primary regression saccades either directly to the target word or at least to the general region of the target, whereas language skill is instrumental in the guidance of additional saccades until the word is successfully attained [12]. Based on the expectation that, in our experiment, regressions to near targets would be much more accurate, we assumed that the relative importance of language skill would manifest particularly in responses to far targets, where the number of necessary saccades should be substantially larger. That is, readers with relatively high scores on static and/or dynamic spatial memory tests may frequently guide the eyes accurately (single shots) to near targets. And, on the other hand, for targets that are relatively far from a current fixation, readers with relatively high scores on a text comprehension test should guide the eyes more effectively to far regression targets than readers with relatively low scores.

## 2. Materials and Methods

### 2.1. Participants

Forty-eight university students (41 female) volunteered or received experimental course credit for their participation and gave written consent prior to participation. Two participants had to be excluded from the data analyses because they did not finish the psychometric assessments for personal reasons. The mean age was 23 years (range = 18 to 34 years). All participants were native German speakers and had normal or corrected to normal vision. The study was approved by the ethics committee of the University of Wuppertal (SK/AE 220829) and complied with the ethical standards of the 1964 Declaration of Helsinki regarding the treatment of human participants. This study was not preregistered. The data were collected in 2018 and 2019. The materials, data and scripts used for the main model analyses can be accessed at https://osf.io/2chn7/ (accessed on 8 September 2025).

### 2.2. Psychometric Assessment

Participants’ reading skill was assessed with the LGVT 6-12 (Lesegeschwindigkeits und -verständnistest, Hogrefe Verlag, Göttingen, Germany), a test for reading fluency and comprehension [21]. It is normed for German students up to 12th grade, when students are approximately 18 years old. It required the reading of text for four minutes. On several locations within the text, three words were presented in parentheses, and participants are instructed to underline the word that was congruent with the context. Two measures, the number of correctly underlined words and the number of read words, were used to index comprehension and speed, respectively (higher values indicated better comprehension and higher reading speed).

Participants’ visuo-spatial working memory was assessed with two subtests of the AGTB 5-12 (Arbeitsgedächtnisbatterie, Hogrefe Verlag, Göttingen, Germany) [22], the matrices test and the Corsi block tapping test (Corsi task). The matrices test started the presentation of a white 4 × 4 grid pattern. On the first trial, 2 squares of the grid were shown in black for 2400 ms. The grids then turned back to white, and participants were asked to touch the grid elements that had been shown in black. After successful completion of a trial, the number of black grid elements was increased by one. The number of black elements remained constant if an error was made, and it was decreased by one—and testing was terminated—when there was more than one consecutive error. The test thus measured an aspect of spatial working memory that represents concurrent (static) spatial locations.

The Corsi task involved the touch-screen presentation of nine spatially separated white boxes on a gray background. A sequence of smileys was then presented one by one (950 ms each) in the boxes. When the sequence was finished, the participant was asked to repeat the sequence from memory by tapping the corresponding order of white screen boxes. The sequence of smileys started with an increase from one to two and was further increased until the participant made a sequencing error. The computer registered correct and incorrect touch screen sequence. Since the to-be-remembered locations emerged over time and had to be reproduced in their correct order, the test was assumed to index the capacity of dynamic spatial working memory. Together, completion of the reading skill and the spatial working memory assessments required approximately 15 min.

The inter-correlation of the four test scores revealed a high positive correlation between the two reading skill components (r = 0.83) and a moderate correlation between the two spatial working memory tests (r = 0.33). The correlations between reading speed and the two spatial working memory scores were low (r = 0.04 and r = −0.07) as was the correlation between comprehension and the two spatial working memory test scores (r = 0.06, r = −0.09).

### 2.3. Reading Task

The to-be-read material consisted of 100 declarative sentences to read. Of these sentences, 64 were defined as experimental sentences and were designed so that the target was located either close to the sentence beginning (far from the probe location) or its ending (near the probe location). If the target word was located at the beginning, it was preceded by two or three words; if it was near the end, it was followed by one or two words. In addition, 32 sentences were used as filler sentences with a regression target near the middle of the sentence. This was used to counteract the development of location specific target expectancies, as the word that matched the probe could have appeared anywhere in a sentence with equal frequency (except for the very beginning and ending).

**Figure 1:**
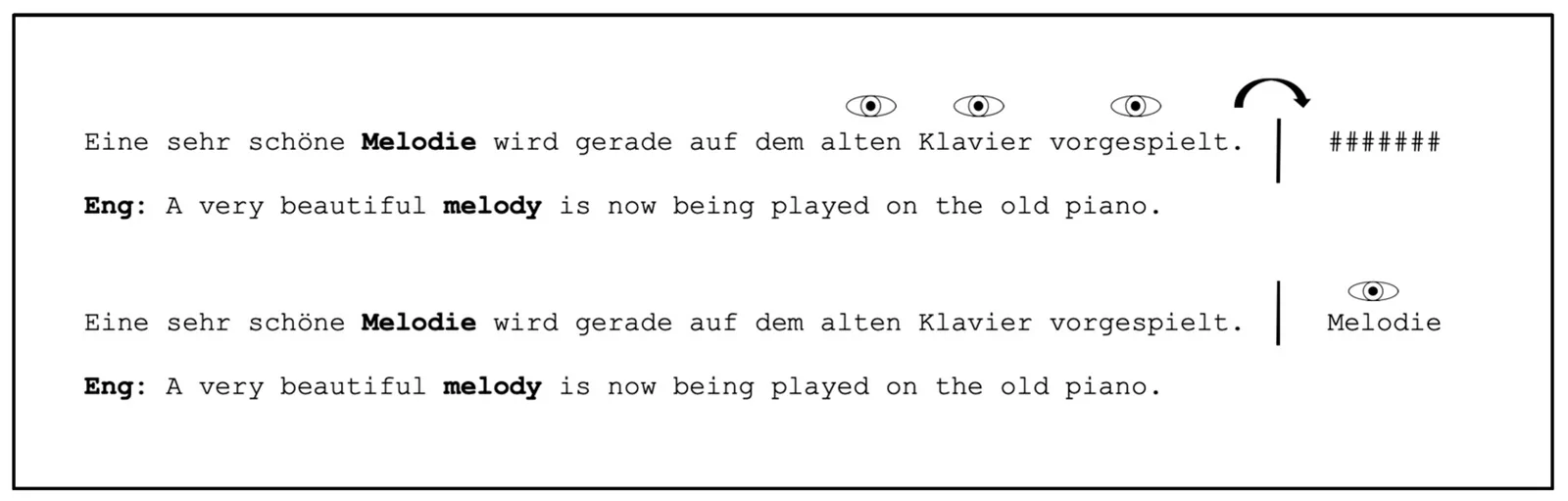
Sentence out of the corpus. Note that the probe word at the end of the line was masked with hash marks and only became visible after participants crossed the invisible boundary. After reading the probe, the corresponding target had to be found in the sentence, to check whether or not it was spelled correctly. Spelling errors were introduced in 50% of the cases, simultaneously with the unmasking of the probe. Highlighting of target words and invisible boundary are for demonstration purposes only. Target words were not marked in any way during the experiment.

An additional probe word—that was identical to the target or filler word in the sentence—was positioned five letter spaces to the right of the last word of the sentence (Figure 1). The probe was masked with a sequence of #s while the sentence was read, and unmasked when the eyes crossed the center of the area between sentence ending and the string of #s (the boundary was placed exactly halfway between the end of the last word in the sentence and the beginning of the probe).

All sentences were 67–70 characters long and contained 9–14 words. All targets (and corresponding probes) were nouns with six or seven constituent letters and either part of the subject, the object or the adverbial complement of the sentence. Target words had a low-medium frequency (mean = 31.1, dlexDB; [23]) and each target word was preceded by an adjective with a length of 5–8 characters.

**Figure 2:**
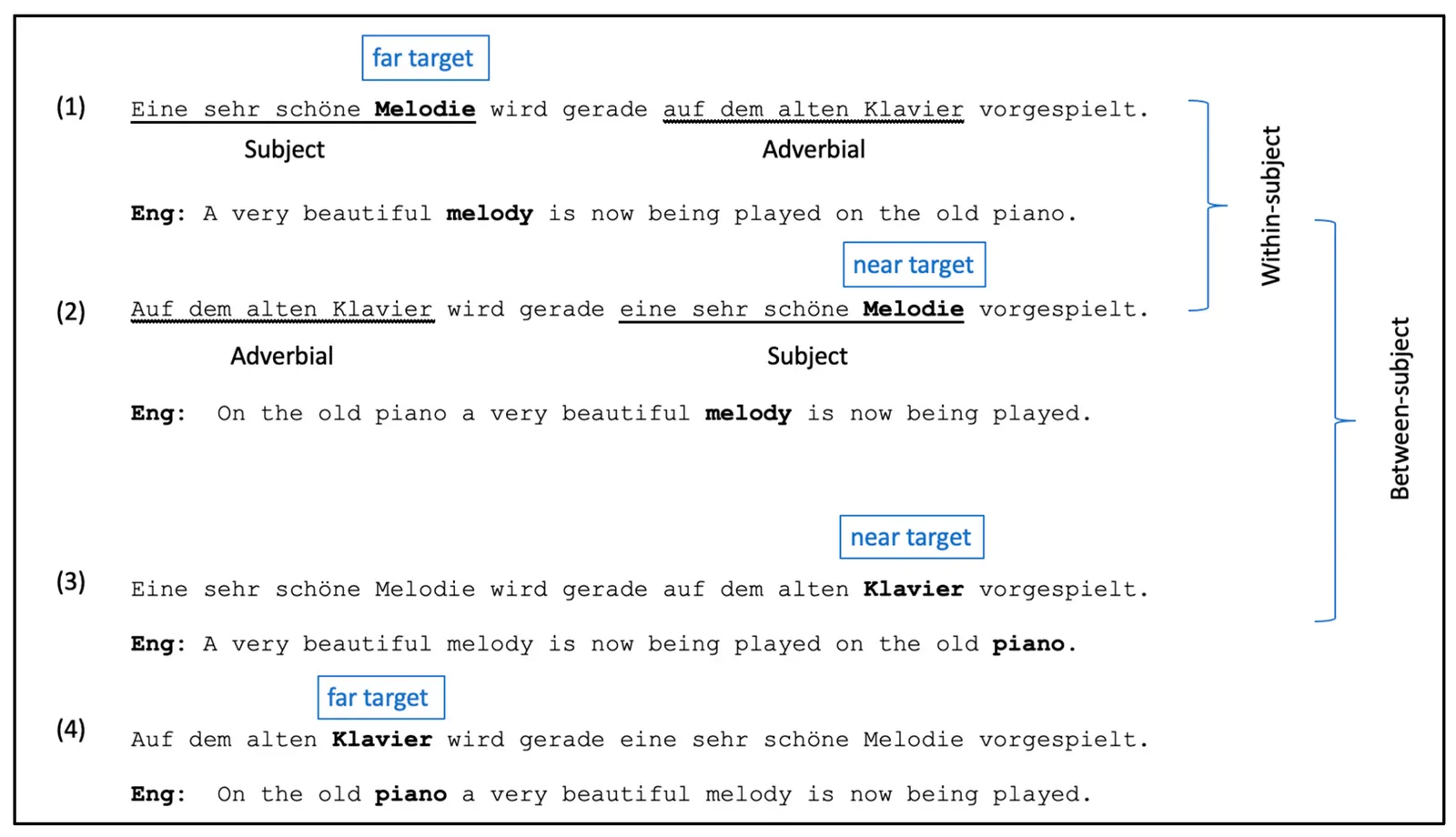
Structure of the sentences. Note that linguistic components are marked for highlighting the German grammar structure. Sentence 1 represents a sentence with a far target (50% of the sentences), which was part of the subject of the sentence. Sentence 2 shows the same sentence frame with the near target position (other 50% of the cases). Sentences 3 and 4 present the same sentence frame, but the target was part of the adverbial construction. Each participant saw every target frame in both distance conditions (i.e., either 1 + 2 or 3 + 4). Each participant also saw 50% of all sentences with a target in the adverbial (Klavier [piano]) and the other 50% in the subject (Melodie [melody]) part of the sentence. All targets were preceded by an adjective with controlled features (see text for details).

German syntax allows the construction of sentences with changes in word order without altering sentence meaning or grammatical well-formedness, as a subject or adverbial phrase can either be the first element or the penultimate element of a sentence. Both alternatives are regularly used and therefore familiar. This linguistic property was used to construct sentence pairs with identical meanings in which the identical target occurred near to and also far from the target. Note that due to this construction rationale, target words could assume different functions within the sentence, for example as the subject, object or adverbial complement, preventing predictions based on syntactic function. In order for each of the 32 target words to appear once at the beginning and once at the end of a sentence, our participants had to read both members of each pair. Figure 2 provides examples of sentences and experimental conditions used in the study.

Similar to Inhoff et al. [13], our design also included a shift manipulation as a supplementary test of the spatial coding hypothesis. In a shift condition, the location of the previously read sentence was shifted one character to the left when the eyes moved to the probe word. No shift occurred in a control condition. If readers used peripheral visible detail to direct regressions, then regressions should be smaller when the sentence was shifted to the left. If, however, regressions were exclusively guided by the retrieval or reconstruction of the target location, then spatial shifting should have no effect.

### 2.4. Procedure

The experiment started with a description of the experimental procedure followed by the sentence-reading task. For this, participants were tested individually. Participants placed their chin and forehead on a forehead rest so that head movements would be minimized and the eye–monitor distance was approximately 68 cm. This was followed by the calibration of the eye tracker and the subsequent presentation of four practice sentences. During practice and experimental (and filler) sentence reading, participants controlled the on- and offset of each sentence with a button press; they were asked to read at a comfortable pace so that they could answer sentence comprehension questions that would occur after the reading of a subset of sentences. These questions were asked after approximately every sixth sentences, and they queried semantic relations (locations, actors, objects, attributes, conditions, causalities) that were described in the sentence. All questions could be answered with a yes or no response, and the correct answer was to be signaled with the pressing of one of the two buttons.

Participants were also instructed to move the eyes to the string of #s that were visible to the right of the sentence ending. They were told that this would reveal a probe word corresponding to a target word in the previously read sentence. To entice readers to execute a regression to the target and to make this task meaningful, they were asked to determine whether the target was correctly or incorrectly spelled when re-read. Half of the targets were unchanged and correctly spelled during re-reading, the other half were misspelled by changing a single non-initial letter of the word.

Please note that all spelling errors were inserted after the sentence was read for the first time, after crossing the invisible boundary. Vowels were replaced by vowels, consonants by consonants and the same category of letters was used to exchange descending, ascending and baseline letters, thus maintaining the original length and word form (e.g., “Melodie” became “Meladie”). These minimal changes were always implemented at near-center locations within words. This ensured that the resulting pronounceable non-words constituted visually non-conspicuous targets for regression saccades. Psychometric assessments took place after the reading task following a break of approximately five minutes. The entire session lasted between 30 and 45 min.

### 2.5. Apparatus

All to-be-read sentences were presented as a single line of text in black font on a light-gray background. Sentences started left-centered with a distance of 400 pixels from the left edge of the screen and were centered vertically in the middle of a 21-inch flat-panel monitor (a resolution of 1680 × 1050 pixels and a refresh rate of 120 Hz). The text was presented in Courier New font, size 15 pt and the viewing distance was set to 68 cm which resulted in a letter width of 0.3° of visual angle. Eye movements were recorded with an SR EyeLink1000^®^ video-based eye-tracking system (SR Research, Toronto, ON, Canada). Viewing was binocular but only the right eye was recorded. The registration is based on infrared light reflection from pupil and cornea at a sampling rate of 2000 Hz. A three-point calibration and validation routine were performed at the beginning of the experiment and after each question to maintain a spatial resolution of approximately 0.3°. In addition, a drift check was carried out before each sentence to ensure accuracy between calibrations. If drift checks indicated deviations greater than 0.3°, an additional calibration was performed. Display changes were initiated when the right eye crossed an invisible vertical line to the right of the sentence (see above) and completed within less than 11 ms.

### 2.6. Oculomotor Measures, Data Selection and Data Analyses

Eye-tracking data were processed using the software suite EyeMap [24]. Since the study sought to examine regression guidance, four regression-related oculomotor measures were extracted and analyzed: (1) initial regression size, consisting of the size of the first saccade (in letter spaces, LS) from the probe toward the target location; (2) the regression error, comprising the distance (in LS) between the initial landing position and the center of the target, (3) the number of regressions (first regression and all following saccades) that were needed to reach the target word after the probe word was fixated; and (4) total regression time, comprising the interval (in ms) between the onset of the first regression toward the target and the fixation of the target.

The eye-tracking records were examined for missing data, and trials with missing data for one or more of the four oculomotor measures were dropped (1.4%). Values of temporal parameters were log-transformed for statistical analyses to better fit normal distributions. To remove outliers, we used the following exclusion criteria: for initial regression size outliers with log values of <2 letter spaces (0.6%), for regression error outliers with log values of <−2 and >4 (1.3%), for number of regressions outliers with log values of >2.5 (1.5%), and for total regression time, outliers with log values of >8 (1.4%) were excluded.

In a first step, the four regression measures from the reading task and the performance in the three assessment tests were analyzed separately to identify significant predictors of readers’ regressions. Individuals’ centered scores were used as continuous variables for statistical modeling. In a subsequent step, this was followed by analyses of the combined regression data and assessment data. A final step comprised a qualitative selection of regression strategies (types) and an examination of regression accuracy as a function of regression strategies. Linear mixed models (LMMs), as implemented in the lmer function from the lme4 package [25,26] in the R environment for statistical computing (R Core Team; version R 4.5.1), were used to analyze the four regressions measures. The LMMs included the fixed effect positions (near vs. far), shift (shift vs. no shift) and interaction of these factors.

Specifically, maximum likelihood comparison revealed that only the variance components of participant and item intercepts and participants’ slope for the position effect improved the model. Participant’s results for the four test measurements (dynamic spatial memory from Corsi test, static spatial memory from matrices task and reading speed and reading comprehension from the reading task) were subsequently added to simplified LMM models which included position, assessment level, and their interaction as fixed factors.

In order to represent the performance of the participants on the assessment tests in a clear way, we decided to define three levels of performance (lower, middle, and upper level) for each of the four tests measures (comprehension, reading speed, matrices memory, Corsi memory). These performance levels were used only for the graphic depiction of data in graphs and tables. The lower-level comprised participants whose performance fell into the lowest quartile (lower 25%), the middle level comprised the two center quartiles (the interquartile range), and the upper level comprised the upper quartile (the upper 25%). The number of participants in each group and percentile ranking of participants in each level are provided in Table 1.

### 2.7. A Classification of Visuomotor Strategies for Regressions

**Figure 3:**
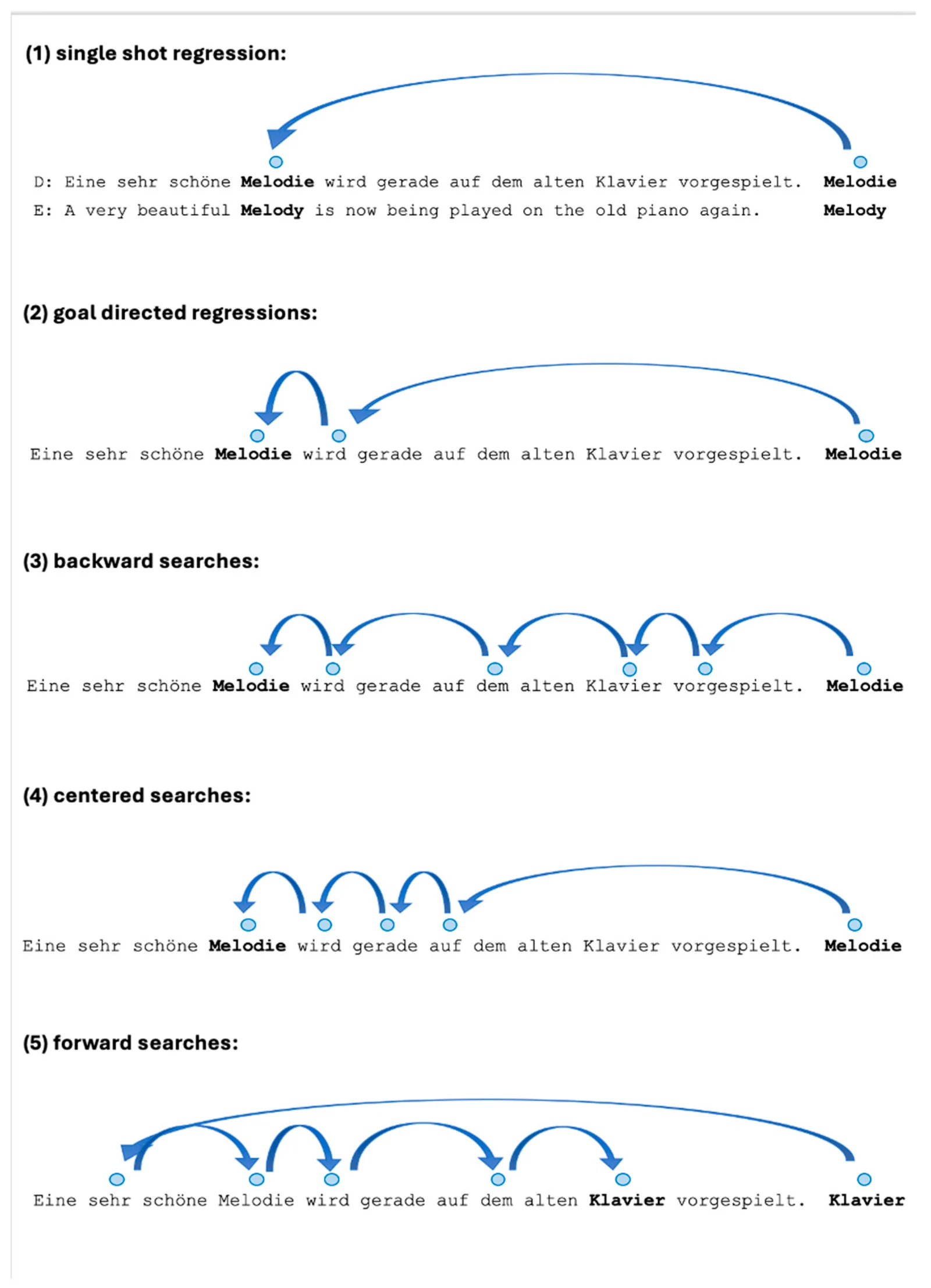
Illustration of the five different regression patterns. Accurate regressions include single shot and goal-directed regressions (with corrections). Search patterns include strategies where at least three saccades are necessary to attain target words. Based on the landing position of the initial regressive saccade, these strategies are divided into backward, centered and forward searches.

According to Murray and Kennedy [10], readers used two types of strategies to reach the regression target: they either moved the eyes with a single regression onto it (single shot regressions), or they missed the target and then scanned backward in the sentence to reach the target (backward search). Visual inspection of regression data in our study indicated a much more varied inventory of qualitatively different search strategies. We propose to classify the visuomotor patterns utilized by readers to attain target words as follows:

(1) single shot regressions, which occurred when a single regression positioned the eyes within a target word (21.3%); (2) goal-directed regressions, which occurred when initial regressions positioned the eyes more than half way toward the target center, and when this was followed by a single smaller saccade to the target (22.2%); (3) backward searches, which occurred when the initial regression was relatively small, landing in the ending third of the sentence, and when this was followed by at least two additional saccades to the target (18%); (4) centered searches, which occurred when the initial regression landed near the middle of the sentence, and when this was followed by least two additional saccades toward the target (28.33%); and (5) forward searches, which occurred when readers executed a long regression to the beginning of the sentence and then executed at least two additional saccades to reach the target (3.3%). The eye movements toward the target were haphazard and unclassifiable on a small number of trails (5.6%); see Figure 3 for a depiction of the classification of regression strategies.

In a subsequent step, we examined whether readers’ modal search strategy for the target was associated with their level of reading skill (comprehension and speed) and/or their spatial working memory (matrix [static], Corsi [dynamic]). For this, participants with an upper or lower level of test performance were selected, and their modal search strategy for the target was determined. When a participant used several types of searches with equal frequency in a particular experimental condition, the weaker search strategy was selected (e.g., a tie between single shot regressions and goal-directed regressions would default to goal-directed regressions and a tie between centered search and backward search to backward search [there were five tied values]). Chi-square tests were then applied to determine whether participants’ modal target search strategy and their level of test performance were associated.

### 2.8. Power Analysis

Since several prior studies have yielded highly reliable effects of the spatial position of a previously read target word on regressions, a medium-to-large standardized effect size can be expected for similar spatial manipulations. To our knowledge, no prior work has examined the influence of individual differences in spatial and verbal ability on the spatial position effect.

Following Brysbaert and Stevens [26], standardized effect sizes for the factor target position and for interactions of position with individuals’ verbal and spatial abilities were defined as the ratio of the difference between condition means and the summed standard deviations of all random factor components of a statistical model. The standardized effect size (d) for position was moderate to large, d = 0.51, whereas the d-values of the interactions of position with individuals’ verbal and spatial ability were relatively small, all d < 0.18. In a subsequent step, we entered d-values and the Variance Partitioning Components from the statistical model into Westfall et al.’s [27] power calculator (https://jakewestfall.shinyapps.io/two_factor_power/, accessed on 4 March 2023) to determine the number of participants and items that would yield a significant difference at the *p* < 0.05 level, assuming a power of 0.8. To obtain a significant effect for position, 18 adult participants and 17 items were needed; for the interaction of position with individual differences, power never reached the desired value of 0.8, regardless of the number of participants and items.

Following Brysbaert and Stevens [26], we also used the powerSim and powerCurve functions from the R library simr to estimate the required number of participants and items to obtain a power of 0.8. The simr functions use Monte Carlo simulations to estimate the relationship between the number of participants and items and power for a particular statistical model. Once more, a significant position effect required relatively few participants and items, whereas the interaction of position with individual differences never attained a power of 0.8.

## 3. Results

### 3.1. Regressing to the Target

The analysis of the four sets of data for the variable initial regression sizes, regression error, number of regressions and total regression time revealed a robust effect of target position. Initial regressions were larger when they were directed to a far than to a near target (b = 0.232, SE = 0.03, t = 7.659, *p* < 0.001). Specifically, the mean initial regression size was 35.9-letter spaces (LSs) for far and 28.8 LS for near targets. However, the difference between initial regressions to near and far targets, 7.1-character spaces, was only a fraction of the actual mean distance between near and far target locations (36-character spaces). The regression error was also profoundly influenced by target distance. When the first regression missed the target, the regression error was 21.5 LS for far targets and 10.4 LS for near targets, which is again a highly significant difference (b = 1.05, SE = 0.127, t = 8.264, *p* < 0.001). The number of regressions and overall search time (total regression time) was also influenced by target distance. More regressions were needed to reach far than near targets (b = 0.413, SE = 0.049, t = 8.335, *p* < 0.001), on average 3.2 regressions for far and 2.3 regressions for near targets. Total regression time amounted to 509 ms for far targets and 330 ms for near targets (b = 0.856, SE = 0.093, t = 9.174, *p* < 0.001).

Target shifting had virtually no effect on regression error (b = −0.04, SE = 0.04, t = −1.034, *p* = 0.303), number of regressions (b = −0.034, SE = 0.019, t = −1.777, *p* = 0.0781) and regression time (b = −0.06, SE = 0.037, t = −1.794, *p* = 0.0759), but initial regression data showed marginal interaction effects of target position and shifting, (b = 0.055, SE = 0.026, t = 2.142, *p* = 0.0323). Numerically, a sentence shift increased the size of the initial regression by about 0.98-character spaces for far targets and decreased initial regression size by 0.68-character spaces for near targets. The absence of a shift effect on regression error, number of regressions and regression time and the small size of the initial regression effect suggests that the influence of the shift on spatial regression targeting was spurious, although some readers could have noted a peripheral brightness change in the shift condition. Consequently, the factor target shift was not included in subsequent analyses that examined the influence of individual differences in reading skill and spatial working memory on regressions to the target (see Table 2).

Four LMMs, using the factors target position and assessment level (for comprehension, reading speed, the matrices test [static spatial working memory], and the Corsi test [dynamic spatial working memory]), revealed that reading speed significantly influenced the time needed to find target words, with faster reading speeds being associated with shorter total regression times, (b = −0.0007, SE = 0.0003, t = −2.055, *p* = 0.045), but had no effect on initial regression size, regression error and number of regressions (*p* > 0.4). The level of reading comprehension had virtually no effect on the regression variables (all ps > 0.1).

**Figure 4:**
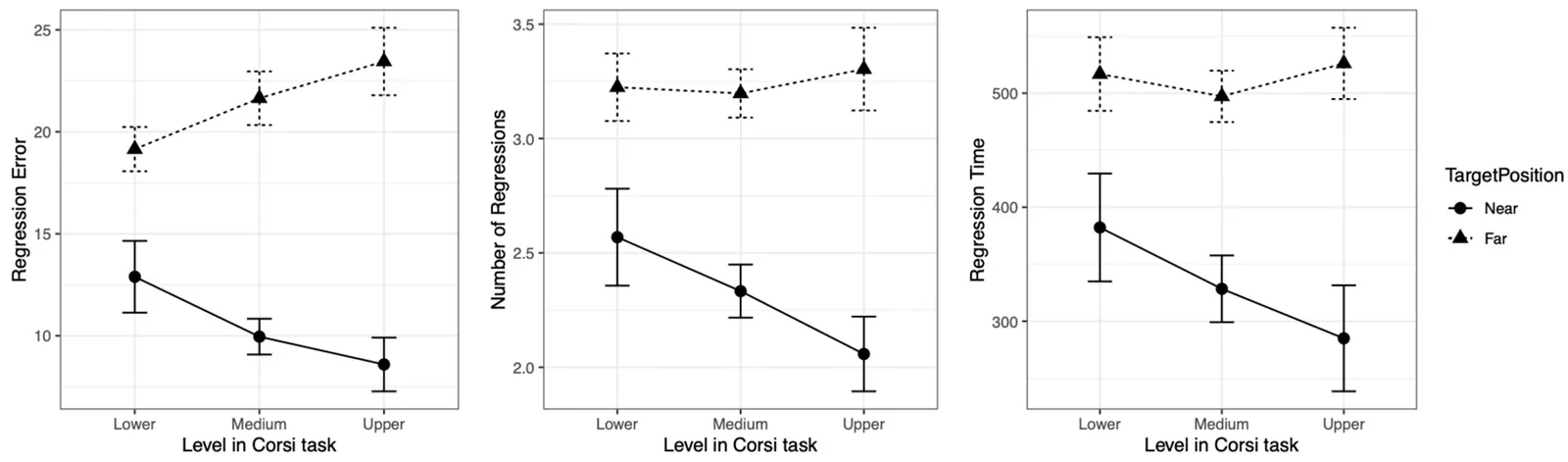
Regression error, number of regressions and total regression time as a function of performance level in the Corsi test measuring visuo-spatial memory in adults. Error bars indicate *SE*.

In contrast to the effects of reading skill, participants’ performance in Corsi test had a profound effect on number of regressions (b = −0.063, SE = 0.029, t = −2.154, *p* = 0.0365) and reading time (b = −0.146, SE = 0.054, t = −2.705, *p* < 0.01), which decreased as test performance increased. Corsi test performance also modulated the target position effect for the three related measure regression errors (b = 0.392, SE = 0.171, t = 2.287, *p* = 0.0269), number of regressions (b = 0.152, SE = 0.064 t = 2.354, *p* = 0.023), and total regression time, (b = 0.307, SE = 0.118, t = 2.597, *p* = 0.0126). Here, increased Corsi performance influenced the three variables primarily for near targets. The corresponding effect pattern for regression error, number of regression and total regression times is shown in Figure 4. Matrices task had no effect on participants’ performance measures with regard to regressions (all ps > 0.1).

Together, the examination of reading skill and of spatial working memory showed that higher comprehension and reading speed had no effect on regression accuracy, but faster reading speed was associated with more effective searches for the target, which often ensued after an initial regression toward the target had been executed. Participants’ dynamic spatial working memory influenced regression number and total search time, but only for near targets.

### 3.2. Interindividual Analyses of Visuomotor Strategies

While the four sets of oculomotor regression data provide quantitative indexes for the reaching of the target, they do not provide a comprehensive picture of how the target was reached and how readers responded when the initial regression did not move the eyes onto the target. Our classification of readers’ search for the target showed that the target’s position had a profound influence on participants’ approach to the target. With near targets, 23 participants used primarily single shot regressions to move the eyes directly onto the target’s location, nine participants preferred goal-directed regressions, and 14 participants preferred centered searches. None of the participants preferred a backward or a forward search for near targets. For far targets, none of the participants preferred single shot regressions, 13 preferred goal-directed regressions, 18 preferred centered searches, 15 preferred backward searches, and none of the participant preferred the forward search strategy.

To examine potential influences of participants’ reading skill and spatial working memory on target search, Chi^2^ tests compared the modal target regression strategy for near and far targets, for participants who scored either in the upper or lower quartile of the four test measures.

**Figure 5:**
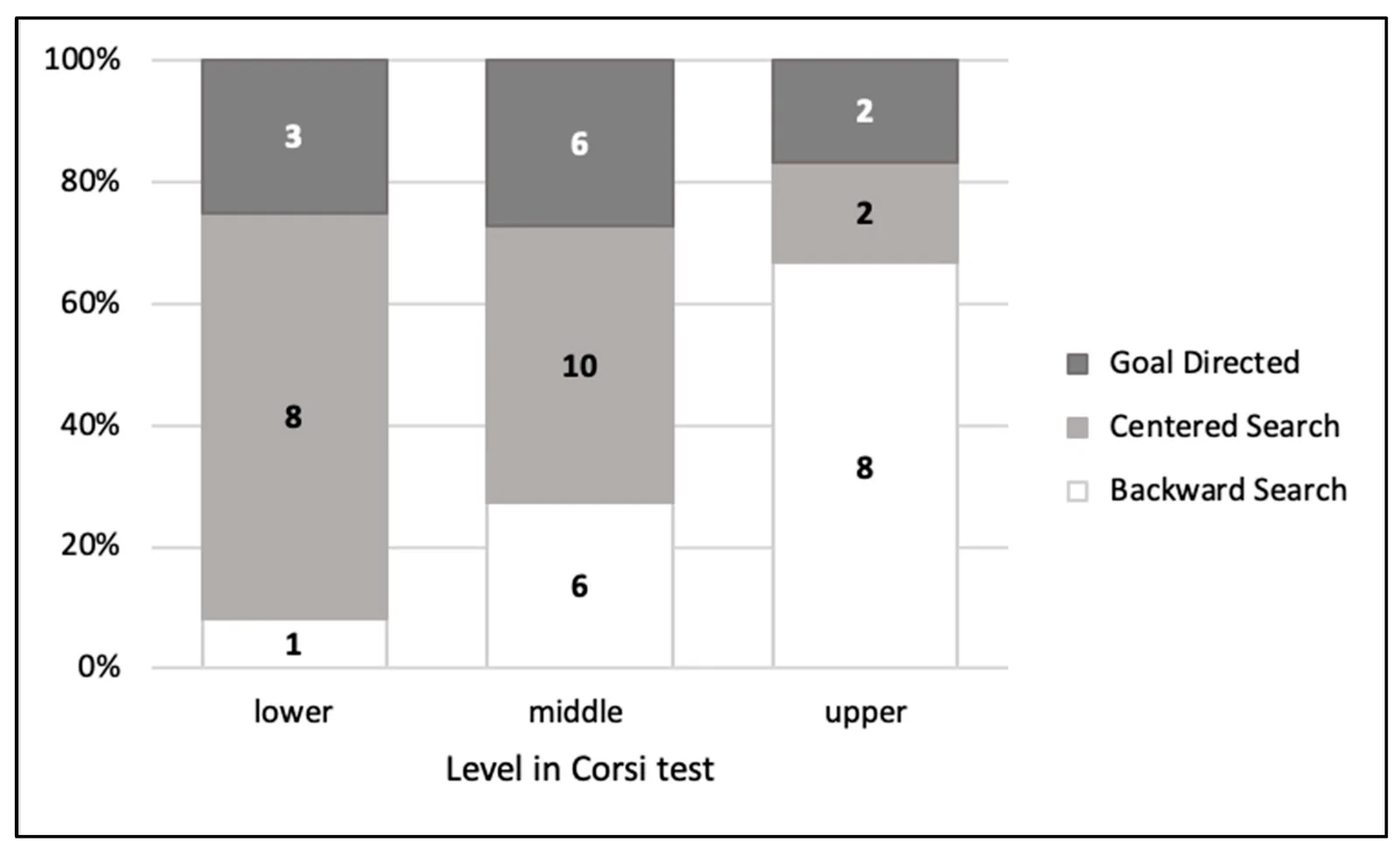
Proportion of preferred used strategies by level of performance in Corsi block tapping test for far targets.

For near targets, the analyses revealed no effect of either reading skills or spatial working memory (all ps. > 0.1). The preferred search for far targets was associated with only one measure, i.e., participants’ Corsi performance, *X*^2^(2) = 9.2444, *p* = 0.009. More individuals with an upper- than a lower-level score used backward searches (eight vs. one, respectively), whereas fewer individuals with an upper-level score used centered searches (two vs. eight), as shown in Figure 5. The backward strategy typically started with an initial regression toward the location that a near target would have had. Had an alternative grammatical sentence construction been used, then this would have been the target’s actual location.

Collectively, these analyses show that participants’ search is influenced by performance in the Corsi test. The search for far targets was influenced only by Corsi test performance, which was linked to the preferred use of backward scanning for high performers.

## 4. Discussion

The present work had two related goals. The first goal was to provide a detailed metrical description of oculomotor behavior used for the re-inspection of (relatively near vs. far) prior words during reading. The second goal was to examine individual differences, focusing on the role of spatial memory and comprehension skill.

In the experimental task, readers were asked to read one-line sentences, and the execution and targeting of regressions was controlled with a probe task. That is, readers regressed back into the sentence from a probe word shown to the right of the sentence ending to re-view a related target word in the sentence. We are aware that our task somewhat deviates from normal reading, in that regressions are elicited after reading each sentence and that the identity of the target word is made clear. However, spelling errors in half of the target words were inserted only after the initial reading. Comprehension questions served to ensure that reading for meaning actually occurred. Most importantly, due to the use of distractor sentences with targets embedded in the center of the line of text, the location of the target word was unpredictable. We believe that the setting used in our work constitutes a good compromise, maintaining normal reading for comprehension, while generating a large number of long-range regressions towards a linguistically specified target word. We see the explicit target specification as the main difference to normal text reading, where the identity of the regression target may sometimes be uncertain. Therefore, we would like to suggest that our results establish an upper boundary of performance that can be expected in completely natural reading.

With respect to the first variable varied in our experimental design, distance to the target, our results were relatively straightforward. Initial regression saccades were spatially selective, being substantially larger when the target word was more distant; see [5] for similar results. However, this disparity was only a fraction of the difference in distance between the saccade launch position and the actual location of near vs. far targets. Consequently, the larger saccadic error relative to the intended saccadic goal was accompanied by a greater number of subsequent saccades until the target was attained. As a result of this larger oculomotor effort, the total time needed to get to the target was much longer in the case of distant targets.

The experimental design also included a sentence shift manipulation to determine whether regressions were indeed guided by information stored in spatial memory or by spatial properties of a peripherally visible sentence. Prior use of shift techniques [8,13] showed that small spatial shifts in either a visual reference frame or the sentence itself did not influence regression size when they were implemented immediately prior to the execution of a target-directed regression. Consistent with these prior results, the consequences of a target shift on initial regressions were negligible in the current study. This finding is in harmony with the general hypothesis that readers utilize stored spatial information for the targeting of long-range regressions.

Looking more closely at the variability in the oculomotor responses to our task, we described two types of successful regression strategies: single shot regressions, as well as goal-directed regressions with small subsequent corrective saccades. In addition, we identified three distinct types of search strategies that were executed when the initial saccade did not land near the target, which we refer to as backward searches, centered searches and forward searches. As we will discuss in more detail below, two of these more complex response patterns had already been found in prior research. One pattern, the centered search strategy, has, to our knowledge, never been described before.

In their seminal analysis of large regressions, Murray and Kennedy [10] distinguished two search types: single shot and backtracking. This was based on the assumption that a successful retrieval of the spatial index of a regression target could be used to reach the target with a single regression. Without the index, readers would search for the target, progressing from the end of a sentence toward the beginning. Examining good and poor readers, the authors found that poor readers were not able to find the required regression target within a sentence and used backward scanning to find the target. Following this, it seemed plausible to expect that readers with upper-level spatial working memory scores would undershoot far targets less than readers with lower-level spatial working memory scores, and that this would yield more backward searches with lower spatial working memory scores.

Going a step further, our design allowed us to relate the preferred use of different visuomotor strategies to individual differences, which was the second goal of the present work. When the target was far, readers with high spatial working memory scores were more likely to direct an initial regression to a near location, and to pursue a backward search strategy to the target. Readers with relatively low spatial working memory capacity, by contrasts, were more likely to direct the initial regression toward the sentence center, unusually undershooting the target. This yielded smaller initial regressions in participants with a high spatial working memory capacity. The centered search strategy may be used when readers could not map their representation of a sentence in phonological onto spatial coordinates, or when the representation of word order was poor. This idea can be seen as a particularly innovative contribution of the present work, especially in conjunction with the functional relation to spatial memory that we have identified.

Our strategy analysis also indicated that a forward search strategy, where readers regress to the beginning of the sentence to re-read it, was rarely used (only 3.3 percent of all cases). In contrast, forward-directed re-reading was quite common in Frazier and Rayner’s [7] analysis of regression strategies after the reading of sentences with garden path constructions. We attribute this to differences in the processing and representation of to-be-read sentences. In the current study, all sentences were relatively easy to understand, and it was unlikely that readers considered sentence processing incomplete or erroneous when the probe word was fixated. Under these conditions, readers preferred represented knowledge to guide the regression back into the sentence. In the (much less common) sentence constructions used by Frazier and Rayner, by contrast, it was likely that readers considered sentence processing incomplete (or erroneous) when the sentence ending was reached, and a forward-directed re-reading may have occurred in order to reparse the words of a sentence.

Our consideration of individual differences revealed that spatial working memory and reading performance made functionally distinct contributions to the reaching of the regression target. Reading performance mainly influenced the efficiency of the correcting process, after the initial regression has been made. Specifically, reading speed determined regression time, with faster readers needing less time to find target words than slower readers. Spatial working memory performance influenced the size of the regression error, indicating an effect on the programming and execution of initial regressions, at least when the target has been read recently (=near position in our experiment). These results are consistent with prior work showing larger initial regressions to far than to near targets, and increases in regression error with increases in target distance (e.g., [13,15,16]). The distinct and apparently sequential contribution of spatial working memory and reading performance also provides converging evidence for Weger and Inhoff’s [12] claim that “initial regressions are primarily guided by spatial memory and rely little on linguistic knowledge, whereas corrective regressions are guided to a much larger extent by linguistic knowledge” (p. 1304). The profound influence of spatial memory performance on near locations was expressed by smaller regression errors, a smaller number of corrective saccades and less overall regression time for high level performer in the Corsi block-tapping task, when attaining near targets. This underlines the particular importance of spatial memory for words that have been read recently.

Long-range regressions adapt to the distance of the target, clearly indicating that retrieval of information from memory takes place and that the retrieved information is utilized to enable re-reading. The advantage for high performers in spatial memory is especially pronounced when regressing to near targets. Far targets were generally more difficult to reach than near targets and the advantage for better spatial performance diminished. It is with far targets, that reading performance could have compensated for poor spatial memory information, explaining the disappearance of the spatial memory effect for far targets. This is in harmony with Guerard et al.’s [15,16] claim, that targeting near and far words may be based on information from different sources of memory. Following their results, verbal memory is the dominant source for the targeting of initial regressions, in particular when the regression target is far, but the underlying process of regression programming to words that have just been read, might differ.

The way of finding a target word changed substantially after the initial regression into the sentence had been executed. Now readers no longer needed to rely on represented forms. Instead, they could extract information from erroneously fixated words and use it to update represented knowledge [28]. The use of new linguistic information may have been more effective for readers with high reading performance, and thus they may have required less re-processing of the sentence to reach a regression target than readers with lower speed level scores.

These accounts suggest that spatial working memory is used to guide initial regressions towards a previously viewed segment of a sentence, as originally suggested by the spatial coding hypothesis [9,29] However, for far targets, spatial working memory does not appear to use accessible spatial indexes of identified words. Instead, it seems to rely on represented word forms to compute an ostensible location for the regression target. Furthermore, the reprocessing of text may be used to direct follow-up saccades when a target location was missing. Linguistic reprocessing can inform the reader where the eyes have landed in the sentence, and whether the meaning conveyed by the target word was expressed earlier or later in the sentence. This information could be used to determine the direction and the size of corrective saccades.

We expect our findings to serve as an important addition to the accumulating body of knowledge on eye movement control during reading. There is a need for more analyses of variability in scanpaths (e.g., [30]). The idea that a spatial representation of word positions is created and that it can be activated and used for the guidance of regressive saccades should also become an integral part of computational models of eye movement control during reading (see [31], for the first attempt in this direction).

## 5. Conclusions

Regressions are spatially selective, being larger for far than for near targets. Far targets lead to longer initial regressions, larger regression errors, but also to more corrective regressions and to a prolonged search time. Our detailed analysis of saccade–fixation patterns revealed that individual responses included five different strategies: single shot regressions, goal-directed regressions (with corrections), centered searches, forward searches, and backward searches. The influence of spatial memory on the regression error size indicates that this is an effective source of programming information for initial regressions, especially when targets are near, i.e., words that have been read recently. Reading performance, by contrast, influenced the subsequent search process, when initial regressions did not attain the target word.

## Figures and Tables

**Table 1:** Participants and percentile ranking by level of test performance.

Level of Performance	Reading Comprehension		Reading Speed		Matrices Test		Corsi Test	
	N	PR	N	PR	N	PR	N	PR
lower	12	9–31	11	9–34	11	12–31	12	12–42
middle	19	39–72	24	36–74	22	38–82	22	54–86
upper	15	84–100	11	76–100	13	84–88	12	95–98

N = number of participants, PR = percentile ranking.

**Table 2:** Means and standard errors of initial regression size, first regression error, number of regressions and total regression time for target position and shift condition.

		Near		Far	
		No Shift	Shift	No Shift	Shift
initial regression size	M	29.17	28.49	35.38	36.35
	SE	0.45	0.45	0.48	0.49
first regression error	M	10.52	10.26	21.83	21.11
	SE	0.37	0.38	0.44	0.44
number of regressions	M	2.38	2.24	3.25	3.19
	SE	0.05	0.05	0.05	0.04
total regression time	M	340.91	318.23	519.05	497.91
	SE	13.19	13.33	11.33	10.30

M = Mean, SE = Standard Error, initial regression size and first regression error are given in character spaces, total regression time in ms.

## Data Availability

The present experiment was part of the doctoral dissertation of Anne Friede, completed at the University of Wuppertal in 2021. The original data presented in the study are openly available in OSF Storage https://osf.io/2chn7/ (accessed on 8 September 2025).
